# *“After all, we are all sick”:* multi-stakeholder understanding of stigma associated with integrated management of HIV, diabetes and hypertension at selected government clinics in Uganda

**DOI:** 10.1186/s12913-022-08959-3

**Published:** 2023-01-09

**Authors:** Mathias Akugizibwe, Flavia Zalwango, Chaka Moreen Namulundu, Ivan Namakoola, Josephine Birungi, Joseph Okebe, Max Bachmann, Murdoch Jamie, Shabbar Jaffar, Marie Claire Van Hout

**Affiliations:** 1 MRC/UVRI and LSHTM, Uganda Research Unit, Entebbe, Uganda; 2 Liverpool School of Tropical Medicine, Liverpool, UK England; 3 University of East Anglia, Norwich, UK England; 4 Kings College London, London, UK England; 5 Liverpool John Moore’s University, Liverpool, UK England

**Keywords:** Stigma, HIV, NCDs, Diabetes, Hypertension, Integrated care

## Abstract

**Background:**

Integrated care is increasingly used to manage chronic conditions. In Uganda, the integration of HIV, diabetes and hypertension care has been piloted, to leverage the advantages of well facilitated and established HIV health care provision structures. This qualitative study aimed to explore HIV stigma dynamics whilst investigating multi-stakeholder perceptions and experiences of providing and receiving integrated management of HIV, diabetes and hypertension at selected government clinics in Central Uganda.

**Methods:**

We adopted a qualitative-observational design. Participants were purposively selected. In-depth interviews were conducted with patients and with health care providers, clinical researchers, policy makers, and representatives from international nongovernmental organizations (NGOs). Focus group discussions were conducted with community members and leaders. Clinical procedures in the integrated care clinic were observed. Data were managed using Nvivo 12 and analyzed thematically.

**Results:**

Triangulated findings revealed diverse multi-stakeholder perceptions around HIV related stigma. Integrated care reduced the frequency with which patients with combinations of HIV, diabetes, hypertension visited health facilities, reduced the associated treatment costs, increased interpersonal relationships among patients and healthcare providers, and increased the capacity of health care providers to manage multiple chronic conditions. Integration reduced stigma through creating opportunities for health education, which allayed patient fears and increased their resolve to enroll for and adhere to treatment. Patients also had an opportunity to offer and receive psycho-social support and coupled with the support they received from healthcare worker. This strengthened patient-patient and provider-patient relationships, which are building blocks of service integration and of HIV stigma reduction. Although the model significantly reduced stigma, it did not eradicate service level challenges and societal discrimination among HIV patients.

**Conclusion:**

The study reveals that, in a low resource setting like Uganda, integration of HIV, diabetes and hypertension care can improve patient experiences of care for multiple chronic conditions, and that integrated clinics may reduce HIV related stigma.

**Supplementary Information:**

The online version contains supplementary material available at 10.1186/s12913-022-08959-3.

## Background

Globally, HIV, diabetes and hypertension are common among adults aged above 25 years [1]. HIV continues to be the leading cause of death among adults in Sub Saharan Africa (SSA) amidst, increasing prevalence of non-communicable diseases (NCDs) [2]. This is largely attributed to increased urbanization that has influenced changes in people’s lifestyles [3]. In South Africa, for example chronic diseases have proved a burden with many patients presenting with multi-morbidities [3, 4].

Increasingly, in low resource settings such as SSA, the costs associated with managing each chronic condition separately are high [3, 5–7]. The integrated care model has gathered momentum as a system of health care provision that manages multiple diseases in the same clinic [6, 8–11]. This model of care has potential for health, social and economic benefits for both the patients and health care providers [12, 13]. It is intended to leverage the well facilitated and established HIV health care provision structures to improve the management of NCDs (enrolment into care, retention and adherence) and reduce associated stigma [9, 14]. However this may sometimes aggravate stigma within the context of chronic diseases management [6, 15]. Integration of services and care can help to de-stigmatize HIV, as well as improving clinical management of comorbidities, and peer support and health education catering for different conditions [16]. The increased uncertainty around future funding for HIV care [17–19] also suggests that integrating NCD care with existing HIV services could be a useful health system strategy. However, where integration has been implemented in Africa, integrated models have been associated with increased numbers of patients, leading to shortage of staff, long waiting times at clinics, poor adherence to appointments and medication, poor confidentiality of medical records, and stigma [6]. Although some studies show that integration reduces stigma associated with HIV [20], others report that integration actually increases community stigma where patients attending the service are assumed to have HIV [10, 14].

In Uganda, the integrated care model has been piloted in selected facilities in Wakiso district in central Uganda in the MOCCA study [8]. The subsequent; Integrating and decentralising diabetes and hypertension services in Africa (INTE-AFRICA) study aimed to support further scale up of the integrated care model of care for NCDs in Uganda. We conducted a qualitative process evaluation [21] to assess the effectiveness and feasibility of large-scale scale up of diabetes and hypertension integration services either on their own or with HIV-services. The study aimed at generating research evidence needed by African health services to scale-up and sustain the screening and management of diabetes and hypertension in different settings. We focus here on illustrating the various dimensions of stigma and dynamics associated with integrated care of HIV, diabetes and hypertension in Ugandan clinics.

## Methods

### Description of the integrated care model

Before integration, HIV care and NCD care were provided separately, by different health workers working in different clinics within each facility. After integration, care was provided in single one-stop clinic in each facility, where patients with at least one of HIV, diabetes or hypertension were managed together. Patients with different conditions were seen and managed by the same clinicians, nurses, counsellors and other staff. There was one pharmacy where medications were dispensed. Patient records for any condition were the same and a similar card was used for all patients. Requisition of laboratory tests for any condition was operationalised in the same place. Integrated clinics were located in primary health care facilities and not in hospitals. Large hospitals were used for referral from lower-level health facilities for complex clinical management. The rationale for integration was that the prevalence of NCDs and of comorbidities with HIV is increasing, that HIV care is well established and that, integration of NCD management with HIV care would help to reduce HIV stigma, support the detection of new cases of NCDs and HIV, and support increased screening diagnosis and adherence to treatment. For this particular study site, we used the former ART clinic building to provide integrated hypertension, diabetes and HIV care.

### Study design

This study had a qualitative-observational design. Participants were recruited from one of the selected clinics where the integrated care clinic was located in Wakiso district in central Uganda. In-depth interviews (IDIs) and focus group discussions (FGDs) were conducted with patients and community members/leaders respectively. Interviews were also conducted with health care providers, policy makers, clinical researchers and representatives from international NGOs. This facilitated an in-depth understanding of their perceptions and experiences regarding how integration of HIV, diabetes and hypertension care impacts on HIV stigma dynamics when such chronic conditions are managed in an integrated service in Uganda.

### Study area and population

The study was conducted at a selected health Centre IV located in a peri-urban area in Wakiso district in Central Uganda. Participants included healthcare users who were enrolled in the integrated care clinic, health care providers, policy makers, representatives from international NGOs and clinical researchers.

### Sampling procedures

Participants were purposively selected. A total of 30 healthcare users who had spent at least 6 months receiving care from the integrated care clinic were purposively sampled in consultation with the study nurse. They were selected according to the disease condition they had, namely; diabetes (DM), hypertension (HTN) or HIV. We purposively selected patients that represented each of the conditions namely; DM, HTN, HIV, and patients that had commodities. Participants in the in-depth interviews were different from those who participated in the focus group discussions. The recruitment of in-depth interview participants was done in consultation with the study nurse who helped to review the patients records and identify patients with different chronic conditions including those with multi-morbidities who had spent at least six months receiving care under integration. In contrast, focus group discussion participants were selected at community level. A village health team member who was also a community leader was consulted and requested to help with the mobilization. These participants were not patients but represented any member from the community surrounding the health facility. Those who accepted to participate in the study were consented and participated. We selected 10 health care providers who were supporting the integrated care clinic. For the participants in the focus group discussions, we recruited five community leaders (both male and female) for each FGD. We also recruited five females and five male community members for the two gender specific FGDs. This number of participants in each FGD was considered appropriate to include a variety of experiences and perspectives, while also enabling each participant to make a substantial contribution to the discussion. The two clinical researchers who were interviewed were part of the INTE-AFRICA trial team. Policy makers and representatives from non-governmental organisations were selected based on their knowledge and experience in offering chronic diseases care management.

### Data collection

Data were collected by two trained social science research assistants (MA and CMN), one male and one female, qualified to Masters level. Data collection was gender matched, with MA interviewing and consulting with males and CMN with females.

Health workers were interviewed in their changing rooms in the clinic while some patients were interviewed in a changing room offered by the health facility in-charge. Some patients preferred to be interviewed outdoors, under the mango tree at the facility. Interviews with policy makers from the government agencies and representatives from NGOs were interviewed using telephones and teleconferencing. The two clinical researchers were interviewed in their offices.

There were no relationships between the participants and the research team before the study. All participants were informed about the goals and reasons for participating in the study at the time of consenting. Written informed consent was obtained for all research participants prior to the interview and all participants that consented, were able to complete the study. Recruitment of study participants was in cognizant of gender for gender specific views, age and cognitive ability of potential participants catered for comprehension of study purpose. Only adults aged 18 years and above, both men and women, and of sound mind were recruited. All questions in the data collection guides were first pilot tested and the necessary amendments in terms of grammar and context were made. All participants were interviewed only once each.

The team observed clinical procedures at the health facility, two weeks after the initial data collection, for about eight hours on one of the days. They specifically observed screening at the triage; sitting arrangements; arrangements at different service points; handling of patients’ medical records; dispensing of drugs; location of the integrated clinic; patients and health care workers’ arrival and departure times (for some health care users and health care providers); and availability of medicine and other supplies. They also listened to informal and formal conversations among patients and health workers.

Interviews with healthcare users and community members/leaders were conducted in Luganda. The interviews and observations were carried out with the help of a topic guide/checklist and were based on a prior scoping review conducted by INTE-AFRICA team members and the process evaluation protocol. The main themes explored were: healthcare seeking experiences; views on integrated models and services; experience of management of HIV, DM and HTN; and lessons learnt and best practices. These were discussed along with sub-themes that emerged. COVID 19 standard operating procedures were observed during all interactions. All interviews and FGDs were audio recorded and the research assistants also wrote separate field notes during the data collection. Individual interviews lasted between 30 min to one hour, while FGDs lasted between one and two hours. Debrief discussions were held every week during which the research assistants presented the key emerging study findings to the rest of the study team. These discussions helped to determine the saturation point where no new insights were realized with addition of more participants.

### Data management and analysis

In this paper, data management, analysis and presentation procedures adhered to Consolidated Criteria for reporting qualitative research. All interviews were transcribed verbatim and patients’ transcripts were translated from Luganda to English by the two research assistants (MA and CMN). No transcripts were returned to participants for comments, data verification or corrections. The team (MA, CMN and FZ) read and re-read the transcripts to identify emerging and recurrent themes which were used to develop a codebook to code the data. The codes were compared for similarities and differences and then grouped into categories. The categorisation was done according to key concepts that emerged from the data, in relation to the study aims. From the grouped categories, themes were then generated by interpreting the categories for their underlying meaning. The themes and sub-themes were transferred into Nvivo 12 management software and analysis was done using a thematic approach. Similar and contrasting opinions from participants were identified [22]. Participants did not give feedback on the study findings. Consistency of data and findings was achieved through backing up the findings with identified corresponding quotations to illustrate participants’ views.

Data safety was ensured by following the MRC/UVRI and LSHTM data protection guidelines and included ensuring transcriptions were encrypted with a password protected code and uploading data to an encrypted sharing point only accessible by the research team. To keep participants’ personal information anonymous, we also used identification numbers to present quotations from the transcripts and deleted the encrypted data afterward.

### Ethical considerations and observation of regulatory guidelines and ethical principles

The study received ethical clearance and approvals from the research ethics committees of the Liverpool School of Tropical Medicine (UK), IS Global (Spain), the National Institute of Medical Research (Tanzania) and Uganda Virus Research Institute Research. The following two Institutional Review Boards including; Uganda National Council for Science and Technology-UCST HS2740, and The Aids Support Organization(TASO)-TASO REC/090/19-UG-REC-009, approved the study in Uganda. To ensure Research ethical procedures and principles including; respect of human as study subjects, autonomy, beneficence and do no harm were observed in accordance with the declaration of Helsinki, Uganda National Council for Science and Technology guidelines were followed and adhered to in all the methods that were conducted [23]. We did not conduct any experiments on humans and neither did we use any of human tissues. Participants were assured of privacy and confidentiality and all and all participants gave written informed consent to participate in the study.

## Results

### Participant profile

Characteristics of participants are shown in Table 1. We interviewed 30 patients of whom 12 (40%) were men and 18 (60%) were women. Most patients had completed senior four (S.4) schooling and none of them had a university degree. At health facility level, of 10 health care workers interviewed, three had a university degree, one had completed primary school (community linkage officer) and the rest had high school certificates (S.4 and S.6) or professional qualifications (midwife, nurse or counsellor).

**Table 1:** Socio-demographic characteristics of study participants

Variable	Participant category							
	**Health care users (patients)**	**Health care providers**	**Community members**	**Community leaders**	**Policy makers**	**Clinical researchers**	**International NGOS**	**Total**
**Age**								
25–29	0	4	5	0	1	0	0	10
30–34	2	1	2	0	0	0	0	5
35–39	4	4	1	2	1	0	0	12
40–44	5	1	1	0	2	1	0	10
45–49	8	0	1	1	1	1	0	12
50 +	11	0	0	2	0	0	1	14
**Sex**								
Men	12	1	5	3	5	1	0	27
Women	18	9	5	2	0	1	1	36
**Education**								
No education	0	0	0	0	0	0	0	0
≤ Primary (P.7)	3	1	0	0	0	0	0	4
≤ Senior four (S.4)	21	3	4	2	0	0	0	30
≤ senior six (S.6)	4	1	3	2	0	0	0	10
Diploma	2	2	1	0	0	0	0	5
Degree	0	3	2	1	5	2	1	14
**Marital status**								
Married	18	4	6	3	5	2	1	39
Single	8	6	3	2	0	0	0	19
Widow/widower	4	0	1	0	0	0	0	5
**Family status**								
Has children	30	8	7	5	5	2	1	58
Has no children	0	2	3	0	0	0	0	5
**Disease condition**								
Had HIV alone,	8	-	-	-	-	-	-	8
Had HTN alone	6	-	-	-	-	-	-	6
Had DM alone	4	-	-	-	-	-	-	4
Had HIV and HTN	3	-	-	-	-	-	-	3
Had DM and HTN	6	-	-	-	-	-	-	6
Had HIV and DM	2	-	-	-	-	-	-	2
Had all conditions	1	-	-	-	-	-	-	1
**All participants**	30	10	10	5	5	2	1	63

Of patients interviewed, eight had HIV alone, six had hypertension alone, four had diabetes alone, three had HIV and hypertension, six had diabetes and hypertension, two had HIV and diabetes and one had all three conditions. Thirteen participants (21%) were aged above 50 years and only five participants were aged between 30–34. In terms of marital status, (61%, *n* = 38) were married, (31%, *n* = 19) were single and five participants (8%) were widowed. In terms of family status, more than (92%, *n* = 57) of participants had children.

### Benefits of integration in chronic diseases management

There was consensus among all stakeholders that integration of HIV and NCDs was an optimal and feasible model for the management of chronic conditions especially in low resource settings.

#### Benefits to patients

The model was perceived to assist patients to reduce the frequency of travelling to the health facilities and associated costs (time and transport), receive screening, diagnosis and treatment of multiple diseases, and receive adequate health education regarding the management of chronic conditions.

*The HIV patients used to feel very uncomfortable to come to receive their treatment. In fact, we used to have fewer patients than we now have for HIV but ever since we brought here the patients with NCDs, we now have registered an increased number of patients who are receiving HIV treatment* **(28-year-old, Female, Nurse)**

*The reason why I say it is good is because before I joined this clinic, I didn’t know that I had diabetes, but when I came here they told me that I also have it so now I am receiving treatment for both hypertension and diabetes and that is good for me otherwise I would have died* **(42-year-old Female, HTN & DM).**

#### Benefits to health care providers

Integration also had benefits for health care providers because they learnt how to manage people with multiple conditions and also had an opportunity to attend to many patients in one consultation, which helped them to have enough time to do other clinical duties.

*This model is working for me because I used to only know how to manage HIV but now I [am able]to test for diabetes and I can advise patients, which is also good for me to broaden my experienc****e* (29-year-old Female, Counsellor)**

*It helps me to properly plan for my weekly activities because I know that in a week I will spare that one day to see the patients for all the three diseases so the rest of the days I’m doing other things. In fact, I’m planning to go back to school because now I have some time to concentrate* **(42-year-old Female, Nursing officer).**

#### Integration improves enrollment in care

Integration was seen as a big opportunity to address stigma associated with enrolling into treatment in the ART clinic. With patients presenting with multiple conditions at the same time and place, individuals with HIV no longer felt singled out and labelled when accessing care from the ART clinic.

*We are likely to increase the number of HIV people receiving HIV care by integrating it with NCD care to take away that labeling and looking at it in that way for me I think it is a bigger opportunity to address stigma than actually the challenge of access to hypertensive [treatment]* **(Male policy maker-1).**

Another participant also revealed that:

*I think stigma among people living with HIV is still there but one way to maximize efficiency is to first of all integrate the services of NCDs within the existing services of HIV. In our main research that we have implemented we found it [Integrating NCDs in HIV care] not to have any aspects of stigma. I mean it has not increased stigma even from the patient’s perspectives we have not found any issues related to stigma* **(Male Policy maker-2)**

Relatedly, another participant argued that the reduction of stigma among patients with HIV may have led to improved nutrition in these patients, which in turn reduced the notion that HIV patients are visibly malnourished. She argued that HIV patients in the integrated care clinic have adhered to recommended feeding practices and are looking much healthier than patients with NCDs who are prohibited from eating certain foods.

*Mmm now it [stigma] has reduced, actually it is not there at all. Because even when you are a diabetic or hypertensive patient, still a person who has HIV is better than you, the HIV patient will be allowed to eat everything and drink everything but you with diabetes, you are stopped from eating or drinking a lot of things, so your immunity is poor compared to a person with HIV. So you find a person with HIV looking very healthy than you with diabetes and hypertension, so how can you laugh at her, in fact it’s you with diabetes and hypertension that should be laughed at for looking unhealthy. But here we don’t have stigmatization because we even don’t know who has HIV and does not have it. Because we all come to the same clinic. Me I see it’s a good method because it has helped a lot to reduce those issues of stigma against people with HIV* **(56-yr-old Female, DM).**

#### Assured privacy and confidentiality

The perception that integration reduces the visibility of HIV stigma within facilities, hiding the individual’s disease identity, was reiterated across a range of participants. This buffered patients from feeling isolated, an experience they reported within the previous routine health care system. In the integrated care model, patients instead talked about now being able to sit together and interact freely.

*We all come and sit together in one place whether you have HIV, diabetes or hypertension, I have no problem with it because, we are all sick. Me I’m very comfortable. You can’t know about the disease unless the person has told you what they are suffering from* **(70-yr-old Female, HTN).**

*I have no problem with it because as you know people who have HIV [used] to stand out a lot, let me put it like that, but if there are many people together, they will not know whether you have come for HIV drugs, hypertension drugs or diabetes drugs* **(46-yr-old Male, HIV).**

#### After all, we are all sick

With patients no longer visibly ‘standing out’ amongst other patients, and with no requirement to disclose one’s condition in waiting rooms, integrated care appeared to provide a space for patients to find common ground in their experiences, as seen here with individuals with HIV and others with DM and HTN:

*No, I do not care, we are all sick. It is just that the conditions we are suffering from are different, but we are all sick. And we all have the same aim of getting drugs* **(47-yr-old Female, HIV).**

*I do not see a problem there unless she is the one who has a problem with it. Because I am taking the drugs for my illness* **(59-yr-old Female, DM &HTN).**

In contrast, shared experience was afforded to some patients through formal counselling, viewed as providing hope as they discovered they were not the only ones with chronic conditions.

*I feel nothing about it because I was counselled and told that I am not the only with the condition. We are many having it* **(47-yr- old Female, DM&HTN).**

*Mmm in our daily life there are some people who call it “mpawo atalikaaba” which means that every person has cried because of HIV, it has reached every family. But me I’m okay with it. I don’t care what they say because I know that I’m not a thief but a patient looking for life support. I talk freely because now I’m used to the people since they already know that I have all the diseases* **(50-yr-old Female, HIV, DM &HTN).**

#### Opportunity for psycho-social support and comfort

Whilst integration enabled patients not to disclose their condition, others reported how integration enabled them to share details about their conditions, providing a platform for mutual support and advice about illness management. In the waiting area, patients exchanged their knowledge and experiences about nutrition, medicines, social support, providing a means of informal counselling. In turn this provided patient –patient relationship and provider-patient relationship.

*Me I feel very free because with these diseases if you don’t open up you will not get medicine to help you. So I talk freely because I have been able to get a lot of support especially in terms of the herbal medicine and the foods that my friends have been able to tell me to use to treat diabetes and hypertension. Because sometimes when we come we don’t get medicine but at least we go back with some advice on how to use herbal medicine to treat the disease* **(42-yr- old Female, DM&HTN).**

*But if we sit together as patients of HIV, diabetes or hypertension, we may counsel each other, talk about the causes of the conditions that we have and you might know what to do to avoid getting the other condition* **(42-yr-old Female, HIV).**

Similarly, during our observation of the clinical procedures, we listened to several patients who shared amongst themselves about drinking and eating bitter herbs and green vegetables, respectively, in order to manage diabetes and hypertension. During such informal conversations, patients gave each other advice and were seen smiling and laughing together. This implied that the integrated care clinic created a sense of unity among the patients. Our observation was related to what one of the participants revealed, that integration enabled them to freely talk about their illness and to get advice from friends;

*I’m very free. Now what do I have to hide, this is a bad disease, if you don’t tell your friends and they give you advise, you can die very quickly. But if you tell them how you are managing and the challenges that you get, they will give you advice* **(56-yr-old Female, DM).**

#### HIV education as part of the integrated care model

Some patients said that the health education provided by the integrated clinic raised their awareness of HIV as a chronic condition. Those who used to think that living with HIV was a death sentence, came to realize that having an NCD could be even more challenging, especially for diabetes where one had to keep taking medicine and sometimes self-inject. This was observed to make patients living with HIV think that they are better off than people living with diabetes. Integration appeared to offer opportunities to learn more about HIV and other chronic conditions.

*In my view, to get HIV you have to have sexual intercourse, so even if we sit together, I will not infect another person, hypertension comes from having too many thoughts, though I am not very sure about that. So it is good if we sit together and talk about our conditions and get us tested because we are not going to infect each other. I do not know what causes diabetes. It is not infectious like COVID-19. You know here people say that you would rather get HIV than diabetes because for diabetes, you always have to inject yourself so you would rather have the one that I have* **(42-yr-old Female, HIV)**

*Treatment for diabetes? What I want is to save my life. I do not think that HIV is a punishment. Back then, they used to tell us that if he/she is talking and his/her saliva falls on you [you get HIV], but HIV of these days is different from the one of those days. I do not think that HIV patients of these days live a sorrowful life like the ones of those days. I know that when we are here, you stop pitying yourself because a person suffering from diabetes like me will die and you who is not will also die* **(48-yr-old Male, DM).**

### Challenges of integration for stigma reduction and chronic illness management

Although the integrated model significantly reduced stigma in general, it did not eradicate service level challenges and societal discrimination among HIV patients. Some policy makers argued that; “*in Uganda, between 2016 and 2019 a lot of work has been done at national, community and individual levels to address stigma and I think stigma generally in Uganda, when you look at the stigma index, is reducing” (****Male Policy maker, 3****).* Despite attributing this reduction to integrated care, they argued that the existing level of stigma still poses a threat, however low it is, and integration was believed to exacerbate stigma, particularly among HIV infected individuals. Accordingly, some patients with diabetes and/or hypertension experienced stigma in the form of “*fear to access services from the integrated care clinic, which is also a former ART clinic”,* concerned that they would be perceived to be living with HIV. This fear was expressed by the majority of participants at the integrated clinic, although those seeking hypertension and diabetes services appeared more at ease than those with HIV alone. Some HIV patients eventually chose to go to other hospitals far from their community in order to feel *‘safe’.*

For some HIV patients, anti-retroviral therapy (ART) seemed to be *‘the thread between life and death’.* In contrast to the reduced visibility observed earlier, some patients reported that they were looked at with pity and were often asked to jump the queue. Such experiences provided a stern reminder that they were surviving on medicine and these favors made them feel stigmatized.

*You go to the clinic and everyone wants you to go and get your medicine because they think that any minute you spend without getting medicine you will die. So people look at you as if you are dying anytime* **(50-yr old Male, HIV).**

Similarly, participants also revealed that labels are still used to describe people living with HIV. Names like; *“silimu”* literally meaning the person is very slim, “*mukenenya*” literally meaning growing thin are used. Others say *“eyo ward ya basiliimu” [that ward is for people who are thin/slim]* **(50-year-old Male, HIV).** Such labels carry stigmatizing messages that deter patients from disclosing their HIV status to those that would support them socially or otherwise.

Health care providers also revealed that initially it was difficult to recruit patients with diabetes and HTN into the current integrated clinic. Diabetic and hypertensive patients did not want to receive treatment with HIV patients saying that; “*at first those[patients] with only HTN and DM had resisted to be managed from that ART clinic, that’s why I’m saying they feared to be recognized with HIV patients…” ***(37-year-old Female, Clinician)**. Similarly; *“some of them declined [joining] the study because of stigma, yeah, that is anticipated also but with time, you know with patients, it is us who keep talking to them and they will understand, yeah”* **(28-year-old Female, Nurse).**

#### What can be done to reduce stigma further?

We draw from the health care providers’ and policy makers’ views that the implementation process of integrated care for NCDs and HIV needs to critically consider the impact of stigma on other aspects of people’s social lives. Some policy makers, on the other hand, perceived the mitigation of HIV stigma to be at the center of chronic diseases management. It is possible to achieve zero stigma if integration is made compulsory for chronic conditions so that no one can single out a patient with a particular disease. Some believed that, “*unless we make it compulsory so that all the patients living with HIV and NCDs begin to receive their treatment in one clinic, we will not achieve the target because HIV is still a highly stigmatized disease in our communities”* **(42-yr-old Male, Policy maker, 2)**. The implication is that patients living with HIV begin to feel comfortable to receive their treatment from the same clinic with other patients who have chronic conditions.

Observation of how the clinical procedures were conducted during the integrated clinic day. revealed that whereas some patients were seen freely talking about their illnesses, others were very quiet. We listened to the kinds of conversations that patients were involved in and discovered that patients talked about how to use herbal medicine to manage NCDs, the recommended food, where to buy prescribed medicine cheaply, among other issues. However patients were careful not to openly discuss HIV related issues. This finding indicated that patients with NCDs were more comfortable to receive treatment in the integrated clinic than patients with HIV were, suggesting that some HIV related stigma persisted.

### Narration of the stigma levels

**Fig. 1:**
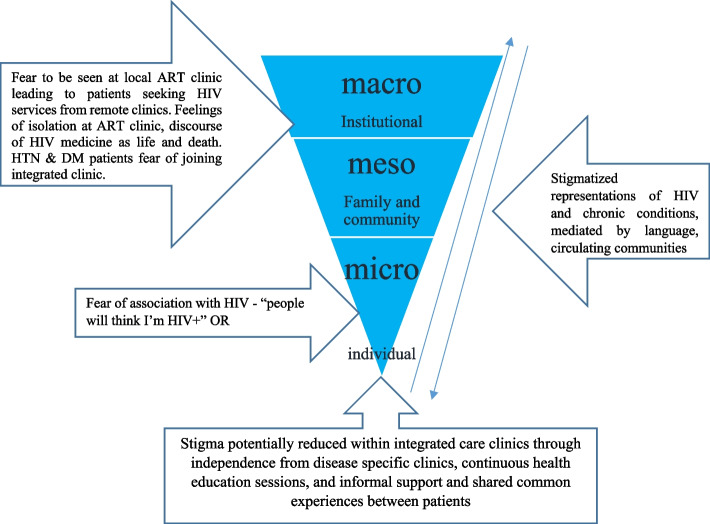
Layers of stigma

Further analysis of our data revealed that stigma presented itself at three levels (Fig. 1). These were: micro (self), meso (interpersonal -family and others) and macro (institutional) levels. At a micro level, some patients were afraid to express themselves and associate with others. Such fears were shaped at a meso level by how different conditions were understood by members of the family or community, mediated through local language discourses, where patients with NCDs were labelled as too old and chronic diseases as death sentences. At the macro level, stigma was in the form of patients with NCDs being afraid to attend facilities in case they were associated with HIV. In this way a cultural discourse of stigma could be seen to circulate across different contextual levels and social spaces, with stigma experienced at the institutional and/or family level informing whether and how the individual disclosed or managed their condition around others. However, because of health education and peer support provided through integration, some patients appeared to have developed a psychological resilience and the sense that they were in control of their conditions.

## Discussion

The study reveals insights into the dynamics and complexities of multi-stakeholder’ perceptions and experiences of providing and receiving integrated care, and insights into how HIV stigma dynamics, and processes of stigma reduction, occur over time.. Our findings re-affirm that integration of NCDs into HIV care is an optimal model in reducing HIV related stigma [8, 19, 24]. In developed countries, integration has been adopted and scaled up to ensure that HIV related stigma is dealt with [25]. This is one reason why integration of NCD with HIV care is being promoted in low resource countries in East Africa [26].

Based on our findings, it is clear that integrating NCDs into HIV care will be a strong pillar in eliminating stigma in the health care system especially for people with chronic conditions. Our findings show that patients seeking HIV treatment felt more comfortable and less stigmatized because of receiving services together with patients with NCDs. This comfort stems from the thought that no one will know which patient has HIV or NCDs since the numbers are many and they are all receiving care from the same service points at the same time. Similarly, it is possible make HIV management more effective, reduce further infections, and reduce stigma if HIV is managed together with other chronic conditions [27]. While at the integrated clinic, patients can hold informal conversations amongst themselves, freely open up about their conditions and seek advice and psycho-social support. Such a benefit shows how integration has improved health education to patients and in return patients no longer see stigma as a problem [8]. However, integrating NCDs into HIV care was to some extent associated with some form of stigma as some patients with NCDs still feared being seen receiving treatment from what had been the HIV clinic building, a phenomenon widely known for offering HIV related services [28]. With integration proving a long awaited move towards elimination of stigma during health care use, the practical implementation and adoption of the model lies in the hands of every stakeholder. Patients and health care providers have already expressed their support for integrated care [29]. A drive for wide-scale implementation of integrated care now requires the backing of policymakers alongside community acceptance.

According to Sullivan and colleagues [30], the Ugandan health care system remains substantially affected by stigma associated with HIV. This dictates the direction that integrated care and management of chronic conditions such as HIV, DM and HTN should take if effective polices and implementation strategies are to be achieved. In our study, stigma was perceived and experienced at all levels (micro, meso and macro) across a range of social settings and spaces. However, stigma was largely attached to HIV than to NCDs. Similar to stigma in leprosy, [31], HIV stigma can be inhibited through sector wide adoption of integration. Whilst integration can take many forms, it is integration of NCDs with HIV care that has shown to be most appropriate in Uganda [32]. Cognizant of these benefits [33], it is time to take advantage of the many opportunities that integration offers, in order to reduce dimensions of social, inter-personal and structural stigma in the health care system.

### Strengths and limitations of the study

Despite our insights into stakeholder experiences of integrated care, we observed that our study was limited to lower level health facilities and used a relatively small sample size which limits generalizability. In addition, given that the former ART clinic building was converted into an integrated care clinic, such an arrangement could have been the reason why facility level stigma was reported. This challenges us that, if the intervention was to be implemented in such a way that an independent clinic is selected other than the ART clinic building, the reported cases of stigma would most likely be reduced further. This would make integrated care optimal in addressing stigma in the management of HIV and NCDs in low resource settings. In order to address some of these limitations, we obtained perspectives from a range of stakeholders and importantly we have identified a breadth and depth of evidence of how stigma was experienced, not only in primary care facilities but within families, communities and the individuals themselves.

## Conclusions

The study builds on a growing evidence base from Sub-Saharan Africa, including Uganda, which supports arguments that integrated care is a plausible and optimal care model for the management of chronic conditions, especially when efforts are channeled to leverage the existing HIV infrastructure and well-funded systems. The study narratives reveal that, in low resource settings like Uganda, integration of HIV, diabetes and hypertension care has the potential to support patient experiences of co-morbid care, and that integrated clinics may function as a central HIV stigma mitigation strategy, operating independently of existing clinics and treating a range of conditions including HIV and other sexually transmitted infections. Integration also enabled patients to have a platform to interact and share amongst themselves about their conditions and experiences of getting treatment, and through such interactions, they offered and received psycho-social support to each other. The model remains vulnerable to HIV related stigma where some patients with NCDs still fear being associated with the clinic where HIV patients attend care, but this is likely to decrease as integrated care becomes standard practice. 

## Supplementary Information

**Additional file 1.** 

## Data Availability

Due to some data in the transcripts containing information attached to the health conditions of the patients that could not completely be anonymized, participants did not consent to having their data publicly availed. However, on request, data to the findings of this study can be accessed from MRC/UVRI and LSHTM social Science server through authorization from the team lead investigator Marie Claire Van Hout. M.C.VanHout@ljmu.ac.uk.
